# Life Skills Education for Malaysian Institutionalised Adolescents: Knowledge, Needs and Priorities: A Qualitative Pilot Study

**Published:** 2017-12

**Authors:** Marjan MOHAMMADZADEH, Hamidin AWANG, Hayati KADIR SHAHAR, Suriani ISMAIL

**Affiliations:** 1. Dept. of Community Health, Faculty of Medicine and Health Sciences, Universiti Putra Malaysia, Serdang, Malaysia; 2. Dept. of Psychiatry, Faculty of Medicine and Health Sciences, Universiti Putra Malaysia, Serdang, Malaysia

## Dear Editor-in-Chief

Life skills education (LSE) is a comprehensive evidence-based plan focusing on the development of the skills that individuals need for a healthy life such as controlling emotions, having effective relationships and life management (1). Indeed, same as any other plans, one of the first components in developing a successful LSE program to respond the special needs of a specific population is the education need analysis (2).

LSE in Malaysia is still at the beginning of its way and there are many potentials for life-skill based program among Malaysian children and adolescents, especially vulnerable ones including institutional adolescents.According to UNICEF, the absence of sufficient life skills to cope with the unique life changes during the period of adolescence, place Malaysian adolescents at higher risk of mental and behavioral problems (3–4). Undoubtedly, the need for LSE among vulnerable Malaysian adolescents, including institutionalized adolescents, is much higher thantheir average peers (5). Therefore, the current study aimed to explore the knowledge, needs, and priorities of life skills among Malaysian institutional adolescents as the first step of developing a sufficient LSE program to improve their mental and behavioral health. Permission to conduct the study was obtained from the ethical committee of the Universiti Putra Malaysia. A written consent form was obtained from all respondents and the caregivers.

Using the Colaizzi’s qualitative research analysis method (6) for collecting data, 31 institutionalized adolescents (18 males and 13 females) from 2 selected orphanages in Klang Valley, Malaysia, were divided into 4 focused discussion groups to discuss their knowledge and needs of life skills. The mean of participants’ age was 14.96 (1.36) and more than half of the participants were male (56.3%). Majority of the participants were Malay (71%) followed by Orang Asli (19.4%) and Indian (9.6%). Secondary school education was selected by all participants as their highest level of education. Most of the adolescents had one alive parent and lived more than 2 years in orphanages.

Discussion sessions (45 to 60 min) were conducted by two local assistants under first author’s supervision. Participants were asked to respond to the following questions:

- Have you ever heard of life skills or life skills education before this study? If yes, tell us about your hearsay.
- Among the life skills explained to you, which one(s) is more important?
- What do you expect to learn in a life skills education session?

Group discussions were continued until reaching data saturation andall of the recording data were transcribed on the same day of group discussions to deduce the main statements and keywords. Prolonged engagement as well as using two data collection methods (focus group discussion and field note), persistent observation and member checks were utilisedtoensurecredibility. Interview transcripts were coded and all participants’ identification factors were removed to ensure confidentiality (7). All data translated from Malay to English once and then translated back to Malay by another researcher. Content analysis and data management were doneusing open coding creating classification and abstraction.

From 31 participants in the study, only 4 adolescents had already heard about life skills (two of them from their teachers in school, one from a television documentary and one from a sharing post on his Facebook page). When the researchersasked them to list the skills categorizing as “the life skills” from their viewpoints, most of the participants mentioned the skills like educational skills, cooking and housekeeping and job skills. From life skills introduced by WHO, only relationship and communication were mentioned by participants. In this stage, in an extra one-hour session, participants received some information about life skills and life skills education by local research assistants. Then, discussion sessions were continued to discover their life skills’ needs and priorities.

The following skills were mentioned by participants as the five most important life skills:

1. Ability to say NO without any hesitance;
2. Ability to control anger and emotions;
3. Relationship skills;
4. Communication and discussion skills;
5. Coping with emotion skills.

Participants also expected to learn or enhance their skills at teamwork activities, the negotiations and conversations, and relationship with their boy/girlfriends during the LSE sessions. A session of LSE better to be informal (not a formal session like school) and full of fun activities such as role plays instead of discussion sessions.

Overall, findings showed the majority of participants have no appropriate knowledge of life skills. Although, after receiving some information about life skills, participants mostly mentioned refusal skills (saying no), commutation skills, anger control and coping with emotions as the most important needed LSE. There is no study explored the life skills knowledge and need among Malaysia adolescents including institutional and non-institutional (8). Due to the importance and intensity of the problems of children and adolescents in orphanages, the findings of this research are intended to helppolicy makers, implementers in health area and orphanages’ caregivers to implement the continuous educational programs based on LSE for institutional and even non-institutional Malaysian children and adolescents in order to improve the public health in the country.
